# Integrating Diagnostic Tools for Early Recognition of Rumenitis in a Neonatal Calf

**DOI:** 10.3390/ani16060870

**Published:** 2026-03-11

**Authors:** Tolulope Grace Ogundipe, Gianfranco Militerno, Riccardo Rinnovati, Raffaele Scarpellini, Talita Bordoni, Arcangelo Gentile, Berihu Gebrekidan Teklehaymanot, Cinzia Benazzi, Marilena Bolcato

**Affiliations:** 1 Department of Veterinary Medical Sciences, Alma Mater Studiorum—University of Bologna, Via Tolara di Sopra, 50, 40064 Ozzano dell’Emilia, BO, Italy; tolulope.ogundipe2@unibo.it (T.G.O.); gianfranco.militerno@unibo.it (G.M.); riccardo.rinnovati2@unibo.it (R.R.); raffaele.scarpellini@unibo.it (R.S.); talita.bordoni2@unibo.it (T.B.); arcangelo.gentile@unibo.it (A.G.); marilena.bolcato2@unibo.it (M.B.); 2 Department of Veterinary Theriogenology and Welfare, College of Veterinary Science, Mekelle University, Mekelle P.O. Box 231, Ethiopia; berihu.gebrekidan@mu.edu.et

**Keywords:** calf, rumenitis, regurgitation, endoscopy, integrated laboratory diagnostics

## Abstract

Rumenitis is an inflammatory condition of the rumen, typically seen in adult cattle managed on high-energy diets. In calves, it is uncommon and often linked to ruminal drinking due to esophageal groove dysfunction. Early diagnosis is challenging due to nonspecific clinical signs. A one-month-old male Limousin calf was presented with persistent non-fetid fluid regurgitation, rhythmic mastication, inappetence, and progressive neurological signs. Clinical examination revealed signs of dehydration and neurological dysfunction. Laboratory evaluation demonstrated metabolic acidosis (pH 7.16), hyperkalemia, and elevated serum urea. Endoscopy identified diffuse mucosal hyperemia, erosions, and fluid accumulation in the rumen. Symptomatic and supportive therapy was initiated; however, the calf died spontaneously. Necropsy was therefore performed, and rumen samples were collected for histological and microbiological investigations. Histopathological analysis confirmed acute suppurative rumenitis. The microbiological culture of rumen and reticulum samples yielded mixed bacterial flora, including *Escherichia coli* and *Proteus mirabilis*. The fungal culture isolated *Penicillium* spp., Mucoraceae, *Geotrichium* spp., and *Aspergillus fumigatus*. This case details the value of integrating clinical examination, blood gas analysis, endoscopy, histopathology, and microbiology in diagnosing rumenitis in young calves. Although Limousin calves are not considered predisposed, management and feeding practices may play a critical role in disease onset. Rumenitis should be considered in calves presenting persistent regurgitation and neurological signs. Early, minimally invasive diagnostics such as endoscopy can improve diagnostic accuracy and inform timely clinical decision-making.

## Simple Summary

Rumenitis is an inflammation of one of the stomach compartments in cattle. It is usually seen in adult animals and is uncommon in very young calves, which makes it difficult to recognize early. In this report, we describe a one-month-old calf that showed weakness, poor appetite, and repeated regurgitation of fluid. Clinical tests revealed serious imbalances in blood chemistry and organ function. An endoscopic examination showed that the inner lining of the rumen was severely irritated and damaged. Laboratory analyses confirmed marked inflammation. Despite treatment, the calf did not survive. This case highlights how quickly rumenitis can progress in young calves and emphasizes the importance of early recognition. It also shows how combining different diagnostic tools, such as clinical examination, blood analysis, and endoscopy, can improve diagnosis and help guide timely treatment decisions.

## 1. Introduction

Rumenitis is a well-recognized inflammatory condition of the ruminal mucosa, often arising secondary to chemical or microbial imbalance. It is most associated with ruminal acidosis in adult cattle fed high-concentrate diets, where the accumulation of volatile fatty acids and lactic acid disrupts mucosal integrity and promotes epithelial necrosis [1,2]. The condition carries clinical significance due to its potential to induce systemic complications, including endotoxemia, metabolic acidosis, and hepatic abscessation [3].

In neonatal and pre-weaned calves, however, rumenitis remains an uncommon diagnosis. Unlike adults, young calves are functionally monogastric, and the rumen is not fully developed until several weeks after birth [4]. Nevertheless, cases of ruminal inflammation in calves have been increasingly linked to ruminal drinking, a pathological condition in which milk enters the rumen instead of bypassing it via the esophageal groove [5]. In previous studies, this misdirection results in milk fermentation within the rumen, generating acid and promoting mucosal damage similar to that observed in carbohydrate-induced acidosis [5,6].

Clinical signs are often nonspecific and may include inappetence, regurgitation, abdominal discomfort, and, in severe cases, systemic or neurological signs related to acid–base imbalance [7]. Diagnosis can be particularly challenging in young animals, where external manifestations may be subtle and laboratory findings difficult to interpret in isolation [8,9]. The integration of endoscopy into diagnostic protocols has allowed for real-time, minimally invasive visualization of the ruminal mucosa, complementing traditional hematological and biochemical analyses [10,11].

This case report describes the clinical presentation, diagnostic workup, and pathological findings in a Limousin calf with confirmed rumenitis, highlighting the role of comprehensive diagnostics, including endoscopy and histopathology, in establishing a definitive diagnosis.

## 2. Materials and Methods

### 2.1. Case Presentation

A one-month-old male Limousin calf presented with a history of persistent regurgitation of non-fetid fluid, rhythmic mastication in the absence of feed intake, inappetence, and progressive neurological impairment.

The herd was managed under a semi-extensive cow–calf system and was vaccinated against enteric diseases and Infectious Bovine Rhinotracheitis (IBR). The calf was dam-raised and received colostrum from the mother; however, the owner chose to administer an additional dose of commercial colostrum via bottle. The calf appeared clinically normal during the first 10 days of life, but subsequently developed diarrhea and was described as dull, frequently recumbent, and reluctant to suckle. Initial treatment included florfenicol and meloxicam for 1 day, followed by oxytetracycline for 3 days, and then paromomycin for 7 days. Concurrently, the calf received vitamin E and selenium, B-complex vitamins, and minerals, administered both systemically and orally. In the days preceding clinical presentation, the regurgitation episodes increased in frequency, and the calf showed signs of intermittent abdominal discomfort and uncoordinated movements.

Upon clinical examination, the calf was in poor body condition with moderate dehydration, evidenced by sunken ocular globes and a prolonged skin tent. Palpable lymph nodes were within normal limits. However, the oral cavity—including the upper and lower lips, palate, sublingual floor, and mucosa—showed widespread papular lesions, some of which were ulcerated. Similar papular lesions were also observed on the muzzle and nostrils. A foul odor was noted.

All other visible mucous membranes and the interdigital spaces appeared normal. No significant findings were noted on the skin and subcutaneous tissues. The temperature was within normal limits, while the heart rate was at 120 beats per minute, and the respiratory rate was 40 breaths per minute.

Among the gastrointestinal and urinary functions, urination and defecation were normal; however, the animal showed a capricious appetite and exhibited episodes of empty chewing followed by regurgitation. Notably, prior to the empty chewing, which closely resembles the rumination motion seen in adult animals, the calf displayed a behavior suggestive of rejecting the mericic bolus, which was in fact expelled, despite the rumen being completely empty upon palpation, poorly mobile, and lacking fermentation sounds. The regurgitated material was liquid, pinkish in color (Supplementary Video S1), with a few straw-like particles and some whitish fibrils. The odor was not acidic. Palpation and percussion with auscultation of the abdomen were negative, both before and after milk administration.

The calf appeared dull and less responsive, with the head either held extended over the neck or lowered. Cranial reflexes and panniculus reflexes, as well as facial sensitivity, were present, though delayed. The swallowing reflex was present, and palpation of both the larynx and esophagus revealed no painful areas, abnormal temperature, or volume changes. Episodes of ataxia, head pressing, kyphotic posture and intermittent lateral recumbency were also noted.

To establish a definitive diagnosis, a full hematological and biochemical workup was initiated, accompanied by venous blood gas (VBG) analysis to assess acid–base balance and systemic compromise. Given the progressive deterioration and suspected ruminal involvement, endoscopy was performed to allow direct visual assessment of the ruminal mucosa and to exclude structural abnormalities. Symptomatic and supportive therapy was initiated; however, after two days of recovery, the calf died spontaneously. During the necropsy, tissue samples from the rumen were collected for histopathological, mycological and bacteriological evaluation to confirm the nature and extent of mucosal lesions.

#### 2.1.1. Blood Sampling and Analysis

The calf was manually restrained in a standing position for clinical assessment and blood collection. Venipuncture of the jugular vein was performed using a sterile 18-gauge hypodermic needle (MicroTip/Ultra, Rays S.p.A., Osimo, Italy) and a Vacuaplast^®^ blood collection system (Aptaca S.p.A., Canelli, Italy). Whole blood samples were collected into EDTA tubes for hematological evaluation and into serum separator tubes for biochemical profiling, including assessment of electrolytes, renal and hepatic function markers, glucose concentration, and inflammatory indicators.

VBG analysis was also conducted to evaluate the acid–base status, respiratory function, and metabolic integrity. Immediately following collection, all samples were transported to the laboratory for prompt analysis using standard automated veterinary analyzers.

Serology for Bovine Viral Diarrhea virus (BVDV) was also performed.

#### 2.1.2. Endoscopic Evaluation

The endoscopic examination was performed under restraint and sedation with xylazine (0.05 mg/kg IV). A 200 cm flexible videoendoscope (Karl Storz^®^, Tuttlingen, Germany) with an external diameter of 9.8 mm was orally introduced. The insertion was facilitated by a mouth speculum to prevent damage to the instrument and oral mucosa. The endoscope was advanced through the esophagus into the rumen under continuous visual guidance, allowing the identification of anatomical landmarks and avoiding excessive insufflation.

Once in the rumen, the mucosal surface was systematically explored under constant air insufflation and low-intensity illumination to enhance visualization. Particular attention was given to assessing the color, vascular pattern, and presence of erosions, ulcers, or adherent material. Images and short video sequences were recorded for documentation and later comparison with histopathological findings. Upon completion, the endoscope was withdrawn slowly to inspect the esophageal mucosa for secondary lesions, and the instrument was disinfected according to manufacturer recommendations.

#### 2.1.3. Gross Pathology

The calf was found dead in the morning, and a necropsy was performed a few hours later. The animal was placed in right lateral recumbency to facilitate systematic access to the thoracic and abdominal cavities and to ensure optimal visualization of the rumen upon opening the abdomen. A standard necropsy approach was used, beginning with an external examination followed by a routine midline and costal incision to expose the abdominal organs. The gastrointestinal tract was inspected in situ before removal, and the forestomachs were opened and examined according to established necropsy protocols.

#### 2.1.4. Histopathological Evaluation

Samples from the rumen, reticulum, and lymph nodes were fixed in 10% buffered formalin for histological examination. Slides were stained with hematoxylin and eosin and Periodic Acid-Schiff (PAS).

#### 2.1.5. Mycological Evaluation

In order to exclude a fungal infection, the biopsy specimens from the rumen and reticulum were processed for cultural examination. In particular, the biopsy specimens were briefly passed through a flame to eliminate any possible surface contamination, then placed on a Petri dish containing Difco ^TM^ Sabouraud Dextrose Agar (BD, Franklin Lakes, NJ, USA) medium with 0.05 g/L chloramphenicol (Sigma, Saint Louis, MO, USA) (SAB-CAF). Three separate ruminal biopsy samples were cultured at both 26 °C and 37 °C. One reticulum sample was cultured at 26 °C and 37 °C.

#### 2.1.6. Bacteriological Evaluation

In order to perform a bacteriological evaluation, sterile swabs with AMIES transport medium were used to collect samples from the oral lesions and the rumen. Swabs were then cultured on media plates for incubation in aerobic (Blood Agar 5% Sheep Blood, CLED Agar, MacConkey Agar) and anaerobic (Columbia Agar) conditions. All the culture media were purchased from a commercial supplier (Oxoid, Milan, Italy). A bacteriological culture was performed by rubbing the swab over approximately one-third of the surface of each media plate and streaking using a sterile 10-microliter loop. After overnight incubation at 37 ± 1 °C, bacterial growth was macroscopically evaluated, and the species identification was assessed using the matrix-assisted laser desorption–ionization time-of-flight mass spectrometry method (MALDI-TOF MS) (Biotyper, Bruker Daltonics, Billerica, MA, USA), following manufacturer’s instructions (Bruker Daltonik, Bremen, Germany), considering an ID score > 2 (green—high accuracy). Identified bacterial species were then subcultured at 37  ±  1 °C for 24 h in Tryptone Soy Agar media plates, and then, Antimicrobial Susceptibility Testing (AST) was performed using the Kirby–Bauer disk diffusion method, according to Clinical and Laboratory Standards Institute (CLSI) guidelines [12]. A total of 7 antimicrobial drugs (Ampicillin 10 µg, Amoxicillin-clavulanic acid 20/10 µg, Cefazolin 30 µg, Ceftiofur 30 µg, Enrofloxacin 5 µg, Tetracycline 30 µg, Trimethoprim-sulfamethoxazole 1.25/23.75 µg) were tested. All the disks were purchased from a commercial supplier (Oxoid, Milan, Italy). For every tested drug, each isolate was classified as susceptible (S), intermediate (I), or resistant (R) based on the 2024 CLSI veterinary breakpoints or, when not specifically present, human ones [13].

## 3. Results

### 3.1. Clinical and Laboratory Findings

Initial clinical examination revealed tachycardia (120 bpm), tachypnea (40 breaths/min), and moderate dehydration. Neurological signs, including ataxia and head pressing, raised the possibility of metabolic encephalopathy, potentially secondary to ruminal dysfunction, electrolyte imbalance, or systemic acidosis. The combination of chronic regurgitation, apparent dysphagia, and neuromuscular incoordination warranted a broad differential diagnosis that included ruminal acidosis with secondary rumenitis, vagal indigestion, metabolic encephalopathy, and possible toxicosis involving the gastrointestinal and central nervous systems.

VBG analysis indicated marked metabolic acidosis, with a blood pH of 7.16 (RI: 7.35–7.45), decreased bicarbonate (HCO_3_^−^) at 19.5 mmol/L (RI: 22–27), and a base excess of −4.1 mmol/L (RI: ±3). A markedly elevated pCO_2_ of 76.1 mmHg (RI: 35–50) indicated concurrent respiratory compensation. Electrolyte analysis showed hyponatremia of 131 mmol/L (RI: 135–145) and hyperkalemia of 4.9 mmol/L (RI: 3.5–4.5), consistent with acid–base imbalance and cellular leakage (Supplementary Table S1).

Serum biochemistry revealed significant azotemia, with urea at 1325 mg/dL (RI: 8–23) and creatinine at 2.04 mg/dL (RI: 0.9–1.3), suggesting renal involvement or severe dehydration. Total bilirubin (0.23 mg/dL; RI: 0–0.1) and indirect bilirubin (0.17 mg/dL) were elevated, reflecting possible hepatic stress or hemolysis. The calf also had hypocholesterolemia (57 mg/dL; RI: 80–120), hypoproteinemia (6.48 g/dL; RI: 6.8–8.6), and hypoglobulinemia (2.76 g/dL; RI: 3–4.9) (Supplementary Table S2).

Following intravenous alkalinizing therapy with sodium bicarbonate, a second blood gas analysis showed partial metabolic correction: pH improved to 7.31, HCO_3_^−^ rose to 28.3 mmol/L, and base excess increased to +5.6 mmol/L. However, clinical signs persisted. The serological test was negative.

#### 3.1.1. Endoscopic Findings

**Figure 1:**
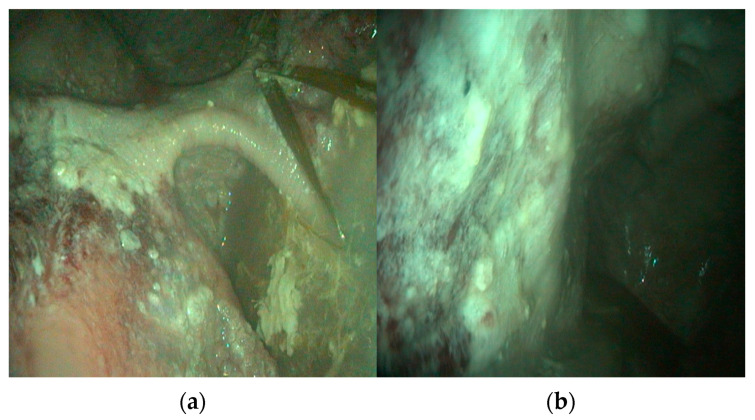
(**a**) Endoscopic view of ulcerative lesion in the dorsal ruminal sac showing mucosal discontinuity and tissue loss, (**b**) endoscopic view of the left ruminal wall showing hyperkeratosis.

Endoscopic examination revealed normal esophageal mucosa in the cervical portion, while the intrathoracic segment showed reddening. The cardia exhibited marked hyperemia. Systematic exploration of the rumen revealed severe pathological changes, with a distinct ulcerative lesion observed in the dorsal sac characterized by mucosal discontinuity and tissue loss (Figure 1a). The left ruminal wall displayed areas of hyperkeratosis appearing as thickened, roughened mucosal surfaces. Throughout the ruminal compartments, the mucosa exhibited a mixed pattern of darker areas consistent with necrotizing tissue and hyperemic zones indicative of active rumenitis (Figure 1b). Multiple rounded lesions with mucosal detachment were distributed across the ruminal wall, showing clear margins and loss of normal papillary structure. No adherent feed material or foreign bodies were observed. Visualization of the reticulum was not possible due to the presence of liquid content.

#### 3.1.2. Gross Pathology and Histopathological Findings

**Figure 2:**
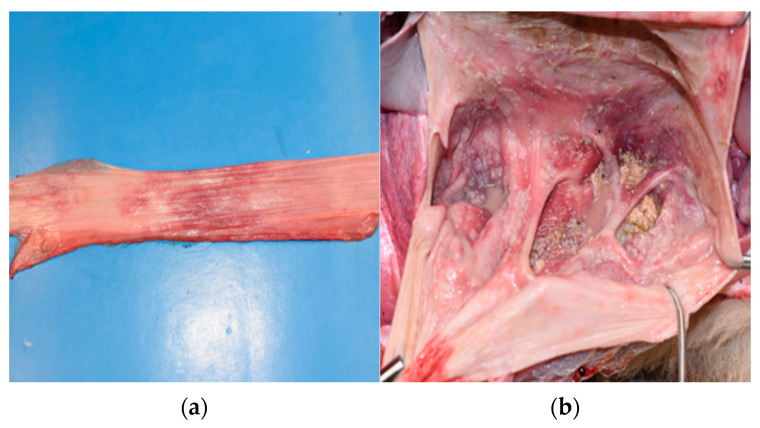
(**a**) Intrathoracic esophageal mucosa displaying hyperemia (erosions not visible in this section); (**b**) acute necrotizing rumenitis showing marked hyperemia and extensive mucosal detachment.

During necropsy, the thoracic portion of the esophagus showed hyperemia with erosion (Figure 2a). The rumen and reticulum showed extensive and severe acute necrotizing rumen-reticulitis, with mucosal detachment and roundish lesions, which raised the suspicion of overlapped mycotic infection by Mucoraceae or other fungi (Figure 2b). No abnormalities were found in the omasum or abomasum. Similarly, the other organs examined (spleen, liver, lung, kidneys, intestine) showed no abnormalities.

Microscopic examination revealed mild diffuse inflammatory reaction of the rumen mucosa and reticulum with infiltration of neutrophils, few lymphocytes and monocytes, thickening of the walls, and no structures attributable to fungal infections (hyphae or spores). In some areas of the sections mucosa and sub-mucosa appeared oedematous, and the mucosa was detached from the inner layers. No evidence of fungi (hyphae or spores) was detected in the PAS-stained sections. In the lymph node sections, an increased number of large follicles, trabeculae and normal lymphocytes were visible.

#### 3.1.3. Mycological Findings

After 48 h of incubation, the plates showed a mixed fungal flora. A macroscopic and microscopic identification was performed. From the reticulum, several colonies of *Penicillium* spp. were isolated at 26 °C, whereas cultures incubated at 37 °C were negative. From the rumen, cultures incubated at 26 °C showed several colonies of Mucoraceae and one colony of a mold presumptively identified as *Geotrichium* spp. At 37 °C, two different colonies of *Aspergillus fumigatus* were isolated, along with one colony belonging to the Mucoraceae.

#### 3.1.4. Bacteriological Finding

All the cultured media from both swabs showed abundant bacterial growth. Two different typologies of colony were macroscopically differentiated and then identified as *Escherichia coli* and *Proteus mirabilis,* both with a high accuracy score. The results of the AST are shown in (Table 1). *E. coli* was found to be resistant to four out of the seven tested drugs, while *P. mirabilis* was found to be resistant to one out of the six tested drugs.

## 4. Discussion

This report highlights a rare and diagnostically challenging presentation of acute necrotizing rumenitis in a one-month-old Limousin calf, characterized by persistent regurgitation, progressive disorientation, and anorexia. While ruminal inflammation is commonly associated with dietary excesses in adult cattle, its occurrence in pre-weaned calves is less frequently documented. In neonatal animals, incomplete closure of the esophageal groove during milk ingestion may result in milk entering the rumen rather than the abomasum [5,14]. Subsequent microbial fermentation of milk substrates can lead to lactic acid accumulation, ruminal acidosis, and progressive mucosal injury. Recurrent episodes of ruminal drinking may also promote chronic epithelial irritation and hyperkeratosis, which was observed endoscopically in this case. Such chronic alterations reduce mucosal barrier integrity and may predispose the rumen to acute necrotizing inflammation following additional stressors, including dietary inconsistencies, systemic illness, or antimicrobial-associated dysbiosis [5,14].

The clinical findings were notable not only for gastrointestinal dysfunction but also for neurological signs such as ataxia, head pressing, and intermittent recumbency. These manifestations, although nonspecific, are compatible with a metabolic encephalopathy secondary to systemic acidosis, endotoxemia, or metabolic toxin accumulation reported in previous studies on human neonates [15,16]. The VBG analysis revealed marked metabolic acidosis (pH 7.16) with elevated lactate levels (7.7 mmol/L), indicating impaired tissue perfusion and a suspected circulatory compromise that was similar to those observed by Castro et al. (2025) [17] in arterial blood gas. This is likely a case of severe ruminal inflammation disrupting mucosal integrity and may permit bacterial translocation and endotoxin absorption into systemic circulation, triggering systemic inflammatory response syndrome (SIRS) [17]. This inflammatory cascade, together with D-lactic acidosis and electrolyte imbalance, may contribute to central nervous system depression and abnormal neurological behavior. Concurrent hyperkalemia and hyponatremia further supported a state of metabolic derangement likely exacerbated by dehydration and prolonged anorexia [15,16]. Thus, the neurological manifestations were most plausibly secondary to systemic metabolic and inflammatory consequences of severe rumenitis rather than primary neurological pathology. A distinguishing feature of this case is the integration of diagnostic modalities, particularly the use of endoscopy as a real-time, minimally invasive method to assess the integrity of the ruminal mucosa [5,10]. The endoscopic findings of hyperkeratosis on the left ruminal wall support the presence of a pre-existing chronic condition, as parakeratosis commonly develops in response to repeated ruminal irritation and predisposes animals to subsequent inflammatory episodes [18]. This pre-existing mucosal damage likely created a susceptible environment, as ruminal acidosis causes mild mucosal inflammation that, when coupled with precipitating factors such as antibiotic use and stress, establishes a high-risk environment for opportunistic bacterial and fungal colonization [19,20]. The extensive necrosis visualized endoscopically correlated closely with the histopathological diagnosis of acute necrotizing rumenitis, demonstrating the value of endoscopy for rapid in vivo assessment of ruminal pathology. Ruminoscopy via oral access allows visual evaluation of mucosal surfaces and has been successfully used to diagnose rumenitis and determine the extent of visible pathological alterations [21,22], though the inability to visualize the reticulum due to liquid content represents a technical limitation of this approach.

The visual identification of diffuse hyperemia, mucosal erosion, and excess intraruminal fluid provided immediate evidence of active inflammation. These findings were subsequently corroborated by a macroscopic and microscopic pathological examination study of rumenitis and ruminal lesions, which revealed acute mucosal necrosis and neutrophilic infiltration, hallmarks of suppurative rumenitis [23,24]. Such alignment between endoscopic and histological findings underscores the value of combining visual and tissue-based diagnostic approaches. Although in this case confirmation was achieved post-mortem, endoscopically guided mucosal biopsy could, in principle, allow ante-mortem histological and microbiological assessment, thereby improving early diagnosis accuracy and clinical decision-making.

Interpretation of the microbiological findings requires careful contextualization. The positive fungal culture in the absence of histopathological evidence of tissue invasion suggests that the isolated fungi most likely represented opportunistic colonizers of necrotic mucosa rather than primary pathogens. The fungal species isolated are commonly present in the environment and may act as opportunistic organisms in the presence of predisposing factors such as mucosal damage, dysbiosis, or systemic compromise [25]. Acidic, necrotic ruminal environments favor fungal proliferation, particularly in devitalized tissue where barrier defenses are compromised. Without microscopic demonstration of hyphal penetration into viable tissue, a diagnosis of primary mycotic rumenitis cannot be supported [26,27]. Furthermore, post-mortem changes in ruminal pH and temperature may facilitate rapid fungal proliferation, and environmental contamination during sampling or laboratory handling cannot be excluded, given the ubiquitous distribution of these organisms [28]. Therefore, fungal isolation in this case should be interpreted as secondary colonization rather than etiological causation. Similarly, the multidrug-resistant *Escherichia coli* isolate must be interpreted with caution. Both isolated bacterial species may form part of the normal digestive microbiota of ruminants. Importantly, the calf had received multiple antimicrobial treatments prior to sampling, which may have exerted selective pressure favoring resistant strains while suppressing susceptible organisms. In particular, prior exposure to oxytetracycline may have contributed to the selection of tetracycline-resistant strains detected in both isolates [29]. In addition, herd-level antimicrobial exposure or environmental dissemination of resistant bacteria within the cow–calf system may have influenced the observed resistance patterns. Such prior therapy could have altered culture results and resistance profiles, potentially masking the original initiating pathogen. It is important to emphasize that antimicrobial resistance does not necessarily correlate with bacterial virulence or confirm a primary etiological role in tissue necrosis. The detection of antimicrobial resistance does not necessarily confirm active infection but highlights the importance of culture-guided therapy and prudent antimicrobial stewardship in food-producing animals [30,31]. Empirical antimicrobial administration, particularly in neonatal calves, may inadvertently complicate microbiological interpretation and contribute to resistance development.

From a breed-specific perspective, Limousin calves are not typically reported to be predisposed to gastrointestinal disorders, suggesting that environmental and management-related factors likely played a more significant role in disease onset. Although no dietary irregularities were documented from the recovery farm, the possibility of esophageal groove dysfunction and ruminal drinking should not be dismissed. Previous studies have implicated early-life feeding errors in the pathogenesis of ruminal acidosis in calves [2,32], which supports this hypothesis.

The outcome in this case was poor, with progressive systemic decline. This case underscores the importance of early metabolic assessment and targeted diagnostic investigation in calves presenting with combined neurological and gastrointestinal signs. Blood gas analysis proved critical in identifying severe metabolic acidosis, while endoscopy allowed rapid in vivo visualization of mucosal pathology. Earlier implementation of such diagnostic tools may facilitate more timely clinical decision-making [10,11,33]. Furthermore, this case emphasizes the need for integrated interpretation of microbiological findings alongside histopathology to distinguish colonization from true infection. Future research should further explore the interplay between early-life feeding management, ruminal microbial dysbiosis, and the role of minimally invasive diagnostics in neonatal rumenitis, with the aim of improving preventive strategies and clinical outcomes.

## 5. Conclusions

This case contributes to the growing understanding of rumenitis in young calves and illustrates how clinical, laboratory, and visual diagnostic data can be triangulated to yield a precise diagnosis. It emphasizes the need for vigilance when calves present with non-specific signs like vomiting or neurological impairment, and it emphasizes the critical role of integrated diagnostics in guiding prognosis and clinical management. Further studies exploring the etiological spectrum of rumenitis in pre-weaned calves, particularly those involving milk fermentation syndromes, would be valuable for informing preventive strategies in both beef and dairy systems [34,35].

## Figures and Tables

**Table 1:** Results of the AST of the two bacterial species (*Escherichia coli* and *Proteus mirabilis*) isolated from the oral and rumen swabs.

Antimicrobial Agent (µg)	*Escherichia coli* Breakpoint (mm)	Observed Diameter (mm)	Interpretation	*Proteus mirabilis* Breakpoint (mm)	Observed Diameter (mm)	Interpretation
Ampicillin (10 µg)	≤13	0	Resistant	≤13	26	Susceptible
Amoxicillin-clavulanic acid (20/10 µg)	≤13	16	Intermediate	≤13	28	Susceptible
Cefazolin (30 µg)	≤19	19	Resistant	≤19	24	Susceptible
Ceftiofur (30 µg)	≤17	27	Susceptible	≤17	28	Susceptible
Enrofloxacin (5 µg)	≤16	29	Susceptible	≤16	28	Susceptible
Tetracycline (30 µg)	≤11	0	Resistant	Intrinsically resistant	NT	Intrinsically resistant
Trimethoprim-sulfamethoxazole (1.25/23.75 µg)	≤10	0	Resistant	≤10	0	Resistant

NT: Not tested due to intrinsic resistance. Intrinsic resistance indicates natural resistance of the bacterial species to the antimicrobial agent, independent of acquired resistance mechanisms.

## Data Availability

All study data are presented in the article.

## Supplementary Materials

The following supporting information can be downloaded at: https://www.mdpi.com/article/10.3390/ani16060870/s1, Table S1: Venous blood gas analysis of the calf at presentation; Table S2: Serum Biochemistry result of the calf and respective reference ranges; Video S1: The animal displays repetitive jaw movements characteristic of rumination.

## Abbreviations

The following abbreviations are used in this manuscript:

EDTA	Ethylenediaminetetraacetic acid
VBG	Venous blood gas
BVDV	Bovine Viral Diarrhea virus
IBR	Infectious Bovine Rhinotracheitis
PAS	Periodic Acid Shiff
SIRS	Systemic inflammatory response syndrome
AST	Antimicrobial Susceptibility Testing
