# Supplementary material for: Integrating Diagnostic Tools for Early Recognition of Rumenitis in a Neonatal Calf
Source: Animals (Basel). 2026 Mar 11;16(6):870. doi: 10.3390/ani16060870 (PMC13023322; doi:10.3390/ani16060870)
Supplement: Supplementary file 1 [file animals-16-00870-s001.zip › Table Sx biochemistry.pdf]

Table S1: Venous blood gas analysis of the calf at presentation

PARAMETER	RESULT&UNIT	REFERENCE INTERVAL
pH	7.165	7.35 … 7.45
pH(st),c	7.338	
Hct,c	42.8 %	
pO2	17.3 mmHg	
pCO2	76.1 mmHg	35 … 50
pH(T),c	7.145	
cH(T),c	71.6 nmol/L	
pO2(T),c	19.3 mmHg	
pCO2(T),c	81.9 mmHg	
ctHb	13.9 g/dL	
sO2	25.5 %	
FO2Hb	25.3 %	
FCOHb	25.3 %	
FHHb	74.0 %	
FMeHb	1.0 %	
K+	4.9 mmol/L	3.5 … 4.5
Na+	131 mmol/L	135 … 145
Ca++	1.04 mmol/L	
Ca++	(7.4) mmol/L	
Cl-	86 mmol/L	
Anion Gap	23.3 mmol/L	
Osmolarity	268.1 mOsm/Kg	
Glucose	6.3 mmol/L	
Lactate	7.7 mmol/L	0.60 ... 2.20
$ctCO_2(p)c$	64.3 Vol%	
$ctHCO_3(p)c$	26.3 mmol/L	
$ctHCO_3(P,st)e$	19.5 mmol/L	22 … 27
ABE,e	– 4.1 mmol/L	±3
SBE,e	–1.4 mmol/L	

Table S2: Serum Biochemistry result of the calf and respective reference ranges

Parameters	Unit Value	Reference interval
**CK**	141 U/l	
**LDH**	851 IU/l	690 ... 1445
**AST**	44 U/l	78 ... 132
**SAP**	87 U/l	
**GGT**	24.1 U/l	
**Total Bilirubin**	0.23 mg/dl	0 … 0.1
**Indirect Bilirubin**	0.17 mg/dl	
**Direct Bilirubin**	0.06 mg/dl	
**Total Cholesterol**	57 mg/dl	80 ... 120
**Glucose**	94 mg/dl	
**Beta-hydroxybutyrate**	0.060 mmol/l	
**Total Protein**	6.48 g/dl	6.8 … 8.6
**Albumin**	3.72 g/dl	
**Globulin**	2.76 g/dl	3 … 4.9
**Albumin/Globulin**	1.35	
**Urea**	1325 mg/dl	8 … 23
**Creatinine**	2.04 mg/dl	0.9 … 1.3
**Phosphorus**	12.35 mg/dl	
**Calcium**	10.2 mg/dl	
**Corrected Calcium**	10.0 mg/dl	
**Ca * P**	126.0	
**Sodium**	133 mEq/l	
**Potassium**	6.6 mEq/l	
**Na/K**	20.0	
**Chloride**	86.0 mEq/l	
**Magnesium**	2.74 mg/dl	
**Uric Acid**	1.47 mg/dl	
**TIBC**	361 µg/dl	
**UIBC**	302 µg/dl	63 ... 186
